# Predicting the Number of Reported Pulmonary Tuberculosis in Guiyang, China, Based on Time Series Analysis Techniques

**DOI:** 10.1155/2022/7828131

**Published:** 2022-10-30

**Authors:** Sheng-xiong Yang, Hong-feng Xu, Yong-jia Mao, Zu-hua Liang, Chun-liu Pan

**Affiliations:** ^1^School of Public Health, The Key Laboratory of Environmental Pollution Monitoring and Disease Control, Ministry of Education, Guizhou Medical University, Guiyang 550025, China; ^2^School of Economics and Management, Guizhou Normal University, Guiyang 550025, China; ^3^Guiyang Center for Disease Control and Prevention, Guiyang 550001, China

## Abstract

Tuberculosis (TB) is one of the world's deadliest infectious disease killers today, and despite China's increasing efforts to prevent and control TB, the TB epidemic is still very serious. In the context of the COVID-19 pandemic, if reliable forecasts of TB epidemic trends can be made, they can help policymakers with early warning and contribute to the prevention and control of TB. In this study, we collected monthly reports of pulmonary tuberculosis (PTB) in Guiyang, China, from January 1, 2010 to December 31, 2020, and monthly meteorological data for the same period, and used LASSO regression to screen four meteorological factors that had an influence on the monthly reports of PTB in Guiyang, including sunshine hours, relative humidity, average atmospheric pressure, and annual highest temperature, of which relative humidity (6-month lag) and average atmospheric pressure (7-month lag) have a lagging effect with the number of TB reports in Guiyang. Based on these data, we constructed ARIMA, Holt-Winters (additive and multiplicative), ARIMAX (with meteorological factors), LSTM, and multivariable LSTM (with meteorological factors). We found that the addition of meteorological factors significantly improved the performance of the time series prediction model, which, after comprehensive consideration, included the ARIMAX (1,1,1) (0,1,2)_12_ model with a lag of 7 months at the average atmospheric pressure, outperforms the other models in terms of both fit (RMSE = 37.570, MAPE = 10.164%, MAE = 28.511) and forecast sensitivity (RMSE = 20.724, MAPE = 6.901%, MAE = 17.306), so the ARIMAX (1,1,1) (0,1,2)_12_ model with a lag of 7 months can be used as a predictor tool for predicting the number of monthly reports of PTB in Guiyang, China.

## 1. Introduction

Tuberculosis (TB) is a chronic infectious disease caused by Mycobacterium tuberculosis (*M.tb*), which is spread mainly through the respiratory tract. *M.tb* can infect various organs throughout the body, with lung infections being the most common, and is the 13th leading cause of death worldwide [1–3]. It is estimated that about 25% of the world's population is infected with *M.tb* [4], and that their lifetime risk of developing TB is as high as 5% to 10% [5], which poses a large threat to human life and health. Despite the tremendous efforts made by countries around the world to prevent and control tuberculosis, it will remain a major public health problem. According to the Global Tuberculosis Report 2021 published by the World Health Organization (WHO) [6], the decline in the global incidence of TB has slowed from previous years, with approximately 9.9 million new cases of TB in 2020 and an incidence rate of 127 per 100,000. It is worrying that the incidence of TB in China turns down to be up in 2020, from 58/100,000 in 2019 to 59/100,000, and becomes the second highest burden of this disease in the world [7]. At the same time, because the COVID-19 pandemic in 2020 has had a huge impact on the provision of basic services for TB, the number of reported TB patients in China has dropped significantly, and about 217,000 cases of TB patients have not been diagnosed or reported to the China Disease Prevention and Control Information System; so, the prevention and control situation is very serious and there is an urgent need to take action to reduce the risk of disease in the population.

PTB is an infectious disease with an incubation period and the potential for widespread population infection [8], and the construction of appropriate predictive models to understand the trend of the epidemic in advance will be of great benefit to the prevention and treatment of tuberculosis. At present, many scholars around the world have constructed mathematical models to be applied in the prediction of infectious disease epidemic trends [9–13], which can be roughly divided into two categories, namely, traditional prediction models and advanced prediction models, and the performance of each method depends on multiple factors, such as data trends, periodicity and noise, and other environmental and social factors, of which the construction of infectious disease prediction models based on time series analysis technology is widely used. In the field of public health and medicine, time series analysis focuses on the continuous observation of biomedical data over time, looking for patterns of change and thus predicting future trends [14]. The ARIMA (Autoregressive Integrated Moving Average) model [15], the Holt-Winters model, and the LSTM (long short-term memory) network model [16] are popular time series models. ARIMA and Holt-Winters models are traditional forecasting models with well-established modeling steps and good statistical properties, but their linear modeling approach suffers from a number of limitations [17]. LSTM models are a type of deep learning and have received widespread attention in the field of infectious disease prediction because they can deal well with the long-term dependence of data and remember the long-term relationships of data in prediction and show great advantages in fitting structurally complex infectious disease data [18, 19]. Since TB has certain transmissible properties as an infectious disease, it is worth discussing whether the LSTM model is necessarily superior to the traditional time series model in terms of prediction.

Since 2020, the COVID-19 epidemic has been raging around the world, and in order to control the epidemic, China implemented an unprecedented social intervention strategy on January 23, 2020 to carry out an emergency response, requiring the whole society to mobilize and advocate good personal prevention, wearing masks, washing hands correctly, and maintaining social distancing as routine measures for the prevention and control of the COVID-19 epidemic. Because COVID-19 and TB are both respiratory infectious diseases and both have similarities in prevention and control methods, the conventional prevention and control strategies implemented during the COVID-19 pandemic may have played a crucial role in reducing the incidence of tuberculosis, and this routine measure will continue for a long time in China. Therefore, in the context of the COVID-19 epidemic, building some more sensitive time-series models for the decline in the number of TB cases will be an interesting question.

Related studies have shown that PTB is a seasonal disease in mainland China, with the epidemic showing a distinct “high in the west and low in the east” regional distribution, with temperate continental, highland, and mountainous climates dominating the western region, which is prone to seasonal increases in PTB [20, 21]. Xiao et al. [22] found that temperature, humidity, wind speed, and sunlight may influence changes in PTB incidence by analyzing the relationship between PTB incidence and meteorological factors in Jinghong, Yunnan Province, southwestern China. The incidence of tuberculosis in Qinghai Province [23] and Guangxi Zhuang Autonomous Region [24] also has different degrees of relationship and lagging effect with some meteorological factors. Therefore, climatic factors play an important role in the onset and transmission of tuberculosis and in predicting the number of tuberculosis cases.

To our knowledge, there are few relevant studies that combine the above time series models with meteorological factors and then predict the number of PTB cases. In this study, based on time series analysis techniques, we developed ARIMA, ARIMAX with meteorological factors, Holt-Winters (additive and multiplicative), LSTM without meteorological factors, and LSTM with meteorological factors, respectively, based on the monthly reported number of TB cases and monthly meteorological data in Guiyang City, Guizhou Province from 2010 to 2020. The six prediction models were compared using mean absolute percentage error (MAPE), root mean square error (RMSE), and mean absolute error (MAE), and the best prediction model was selected.

## 2. Materials and Methods

### 2.1. Research Area

Guiyang is the capital of Guizhou Province, located in the eastern part of the Yunnan-Guizhou Plateau in southwest China, between 106°07′ and 107°17′ East longitude and 26°11′ and 26°55′ North latitude, with a total area of 8,034 km^2^ and a resident population of 5,987,000. Guiyang City has a humid and mild subtropical climate with both plateau and monsoon characteristics. The annual average temperature is 15.3°C, the annual average relative humidity is 77%, the annual average total precipitation is 1129.5 mm, the annual average sunshine hours is 1148.3 hours, and the ultraviolet intensity is weak.

### 2.2. Data Scores

Monthly reports of tuberculosis in Guiyang City from January 1, 2010 to December 31, 2020 were obtained from the *Tuberculosis Management Information System of the China Disease Prevention and Control Information System*, which included a total of 38,835 cases of tuberculosis. Meteorological data for the study period were obtained from the *Guiyang City Statistical Yearbook*. Meteorological indicators included average temperature (°C), total precipitation (mm), average atmospheric pressure (100 Pa), relative humidity (%) sunshine hours (hour), annual highest temperature (°C), and annual lowest temperature (°C). Data from 1 January 2010 to 31 December 2019 were defined as the training set to build and compare the prediction models, and data from 1 January 2020 to 31 December 2020 were the number of monthly PTB reports (defined as the test set) in Guiyang City after the onset of the COVID-19 to evaluate the prediction performance of the models.

### 2.3. Variance Inflation Factor

The variance inflation factor (VIF) is a measure of the severity of multicollinearity in a multiple linear regression model. It represents the ratio of the variance of the estimated regression coefficients to the variance of the independent variables assuming that there is no linear correlation between them. The formula is as follows [25]:

(1) $$\begin{array}{c} \text{VIF}=\frac{1}{1-{R_{i}}^{2}}, \end{array}$$

where *R*_*i*_ is the coefficient of determination of the independent variable *x*_*i*_ on the rest of the independent variables doing the regression analysis. The larger the VIF, the stronger the collinearity between the independent variables. In general, if the VIF > 10, it means that the regression model has severe multicollinearity.

### 2.4. LASSO Regression

LASSO, known as the least absolute shrinkage and selection operator, was first proposed by Tibshirani [26] in 1996 and is a popular penalty regression method, which penalizes the absolute value of each regression coefficient by constructing a penalty function so that the regression coefficient is compressed even to zero, while requiring the sum of the absolute values of all regression coefficients to be less than or equal to the penalty parameter *λ* (*λ* ≥ 0). A more refined model is finally obtained. It retains the advantages of subset shrinkage and is a kind of biased estimation for dealing with covariance data and therefore possesses very good results for the treatment of high-dimensional data and the selection of variables. The formula is as follows:

The multiple linear regression equation is assumed to be

(2) $$\begin{array}{c} y=\beta_{0}+\beta_{1}x_{1}+\beta_{2}x_{2}+\cdots +\beta_{p}x_{p}+\varepsilon \varepsilon \sim N\left(0,\sigma^{2}\right). \end{array}$$

The regression coefficients satisfy

(3) $$\begin{array}{c} \overset{p}{\underset{j=1}{\sum }}\left|\beta_{j}\right|\leq \lambda . \end{array}$$

Minimize the sum of squared residuals under (3):

(4) $$\begin{array}{c} {\left(\overset{\wedge }{\beta }\right)}^{\text{LASSO}}=\arg\min\left\{\overset{n}{\underset{i=1}{\sum }}{\left(y_{i}-\overset{p}{\underset{j=1}{\sum }}\beta_{i}x_{ij}\right)}^{2}\right\}. \end{array}$$

In LASSO regression, the *C*_*p*_ (Mallow's *C*_*p*_) statistic is usually used to select the optimal subset, with a smaller *C*_*p*_ value representing the optimal subset.

### 2.5. ARIMA Model

The general abbreviation of the ARIMA model is ARIMA (*p*, *d*, *q*) (*P*, *D*, *Q*)_*S*_ [15], of which *p* represents the autoregressive order, *d* represents the difference order, *q* represents the moving average order, *P* represents the season the order of autoregression, *D* is the order of seasonal difference, *Q* is the order of seasonal moving average, and *S* is the cycle step. The ARIMAX model is an extension of the ARIMA model, and when the ARIMA model includes other time series (meteorological factors) as input variables, it is called the ARIMAX model. The modeling steps for both models are roughly as follows: (1) stationarity and white noise test of the original sequence: use the augmented Dickey-Fuller (ADF) test to judge whether the original sequence is stationary, if it is a nonstationary sequence, then perform sequence transformations, such as logarithmization, difference (*d*), and seasonal difference (*D*), and then use “Ljung-Box” statistic to perform white noise test on the stationary sequence to see if it has analytical value; (2) model identification: the model is automatically identified by the “auto. arima ()” function in RStudio, and the possibility of *p*, *q*, *P*, *Q*, and *S* is preliminarily determined by combining the properties of the stationary series sample autocorrelation coefficient (ACF) and partial autocorrelation coefficient (PACF); (3) parameter estimation: after the model is identified, the value of the unknown parameter in the model is estimated by using the observed value of the sequence. The method chosen for this paper is conditional least squares and maximum likelihood estimation hybrid method (CSS-ML); and (4) model test: the best model is selected by comprehensive consideration of AIC (Akaike Information Criterion), and the residual sequence of the model should be a white noise sequence.

### 2.6. Holt-Winters Model

The Holt-Winters method [27] is a time series analysis and forecasting method divided into an additive model and a multiplicative model. The method is applicable to nonstationary series containing linear trends and periodic fluctuations and uses exponential smoothing (EMA) to allow the model parameters to continuously adapt to changes in the nonstationary series and to provide short-term forecasts of future trends.

The Holt-Winters additive model can be expressed as follows:

(5) $$\begin{array}{c} x_{t}=a\left(t-1\right)+b\left(t\right)+c\left(t\right). \end{array}$$

The recursion formula is as follows:

(6) $$\begin{array}{c} \hat{a}\left(t\right)=\alpha \left[x_{t}-c\left(t-m\right)\right]+\left(1-\alpha \right)\left[\hat{a}\left(t-1\right)+\hat{b}\left(t-1\right)\right], \end{array}$$

(7) $$\begin{array}{c} \hat{b}\left(t\right)=\beta \left[\hat{a}\left(t\right)-\hat{a}\left(t-1\right)\right]+\left(1-\beta \right)\hat{b}\left(t-1\right), \end{array}$$

(8) $$\begin{array}{c} \hat{c}\left(t\right)=\gamma \left[x_{t}-\hat{a}\left(t\right)\right]+\left(1-\gamma \right)c\left(t-m\right). \end{array}$$

The predicted value for the forward *k* period is

(9) $$\begin{array}{c} {\hat{x}}_{t+k}=\hat{a}\left(t\right)+\hat{b}\left(t\right)k+\hat{c}\left(t+k\right),\forall k\geq 1. \end{array}$$

The Holt-Winters multiplicative model can be expressed as follows:

(10) $$\begin{array}{c} x_{t}=\left[a\left(t-1\right)+b\left(t\right)\right]c\left(t\right). \end{array}$$

The recursion formula is as follows:

(11) $$\begin{array}{c} \hat{a}\left(t\right)=\alpha \left[\frac{x_{t}}{c}\left(t-m\right)\right]+\left(1-\alpha \right)\left[\hat{a}\left(t-1\right)+\hat{b}\left(t-1\right)\right], \end{array}$$

(12) $$\begin{array}{c} \hat{b}\left(t\right)=\beta \left[\hat{a}\left(t\right)-\hat{a}\left(t-1\right)\right]+\left(1-\beta \right)\hat{b}\left(t-1\right), \end{array}$$

(13) $$\begin{array}{c} \hat{c}\left(t\right)=\gamma \left[\frac{x_{t}}{\hat{a}\left(t\right)}\right]+\left(1-\gamma \right)c\left(t-m\right). \end{array}$$

The predicted value for the forward *k* period is

(14) $$\begin{array}{c} {\hat{x}}_{t+k}=\left[\hat{a}\left(t\right)+\hat{b}\left(t\right)k\right]\hat{c}\left(t+k\right),\forall \geq 1. \end{array}$$

In the above equation, *a* (*t* − 1) is the unbiased estimate of the sequence intercept term, where the  *t* − 1 moment eliminates the seasonal effect,  *b* (*t*) is the unbiased estimate of the slope of the *t*-moment *b*, *c* (*t*) is the unbiased estimate of the *t*-time seasonal index *Sj*, *xt* is the latest observation obtained at the *t*-time, and *m* is the period length of the seasonal effect; *α*, *β*, *γ* are smoothing coefficients that meet the 0 < *α*, *β*, *γ* < 1.

### 2.7. LSTM Model

The LSTM model, known as the long short-term memory model, was first proposed in 1997 by Hochreiter and Schmidhuber [16]. Because of its unique design structure, it is suitable for handling and predicting important events with long intervals and delays in time series. Currently, application areas include text generation [28], speech recognition [29], machine translation [30], and infectious disease prediction [31]. LSTM is a special kind of recurrent neural network (RNN) [32], which combines short-term and long-term memory through gate control, overcomes the gradient disappearance or gradient explosion of traditional RNN models, and is better at dealing with the problem of multiple variables. The individual circulatory structures of LSTM (also known as cells) consist of input gates, forgetting gates, output gates, and unit states. The output gate determines how much of the input data of the network needs to be saved to the cell state at the current moment; the forgetting gate determines how much of the unit state at the previous moment needs to be retained at the current moment; an output gate is a control of how much of the current unit state needs to be output to the current output value. Prediction methods that use only a single piece of data as input belong to univariatable LSTM and multivariable predictions can be constructed to improve the accuracy of predictions, taking into account that some variables exhibit periodic changes. In this paper, a multivariable LSTM prediction model of PTB reporting was established using the “torch” package in RStudio, which not only considered multiple meteorological elements but also the lagging effect of meteorological factors on the incidence of PTB. Since the LSTM model is sensitive to the input size of the data, the data should be readjusted to the range of 0 to 1 (also known as standardization), and this study has been parameterized several times to determine the optimal model based on the minimum root mean square error (RMSE) of the test set. The main parameters are shown in Table 1.

### 2.8. Model Evaluation Index

The performance and prediction accuracy of ARIMA model, ARIMAX model, Holt-Winters (additive and multiplicative) model, LSTM model, and multivariable LSTM model were evaluated using mean absolute percentage error (MAPE), root mean square error (RMSE), and average absolute error (MAE). The calculation formula is as follows:

(15) $$\begin{array}{c} \text{MAPE}=\frac{1}{n}\overset{n}{\underset{y=1}{\sum }}\left|\frac{\text{actual}\left(y\right)-\text{forecast}\left(y\right)}{\text{actual}\left(y\right)}\right|\times 100\%, \end{array}$$

(16) $$\begin{array}{c} \text{RMSE}=\sqrt{\frac{\sum_{y=1}^{n}{\left|\text{actual}\left(y\right)-\text{forecast}\left(y\right)\right|}^{2}}{n}}, \end{array}$$

(17) $$\begin{array}{c} \text{MAE}=\frac{1}{n}\overset{n}{\underset{y=1}{\sum }}\left|\text{actual}\left(y\right)-\text{forecast}\left(y\right)\right|. \end{array}$$

*y* = 1, 2, 3, ⋯, *n*, and *n* represents the sequence sample size.

### 2.9. Data Processing and Analysis

The number of monthly PTB reports and monthly meteorological indicators in Guiyang City from 2010 to 2020 was collated and summarized through Excel 2010, using the “forecast,” “tseries,” and “torch” packages in RStudio (https://www.rstudio.com/) to establish ARIMA models, ARIMAX models, Holt-Winters (additive and multiplicative) model, LSTM model, and multivariable LSTM model. The statistical test level is *α* = 0.05.

## 3. Results

### 3.1. Epidemic Situation of Pulmonary Tuberculosis in Guiyang

**Figure 1:**
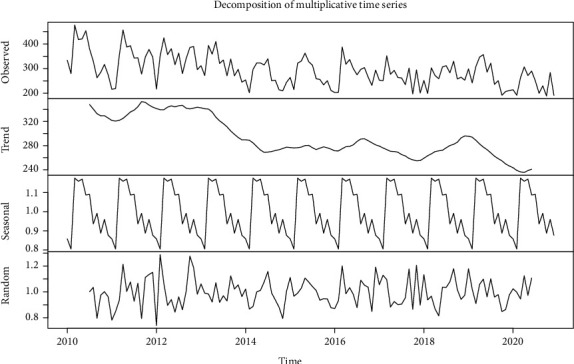
Decomposition of multiplicative time series of monthly pulmonary tuberculosis registrations in Guiyang from 2010 to 2020.

From January 1, 2010 to December 31, 2020, a total of 38,835 cases of PTB were registered in Guiyang City, with the maximum number of monthly reports being 477 cases and the minimum number of monthly reports being 189 cases. From the decomposition of multiplicative time series (Figure 1), there is a long-term trend and a clear seasonality in the number of monthly reports of PTB in Guiyang. The long-term trend shows that the number of PTB reports in Guiyang from 2010 to 2020 shows an overall downward trend. The seasonality shows that the number of PTB reported in Guiyang is the lowest level in February and the high incidence period from March to July and two “small peaks” in September and November. Random effects showed that when trend effects and seasonal effects were excluded, the monthly reports of PTB in Guiyang showed randomness.

### 3.2. Influence of Meteorological Factors on Pulmonary Tuberculosis Registration in Guiyang

From 2010 to 2020, the total precipitation in Guiyang was 99.902 mm (6.300 mm~507.100 mm), the average temperature was 14.752°C (-1.500°C~24.300°C), the average sunshine hours was 86.814 hours (2.400 hours~218.600 hours), the relative humidity was 81.167% (68.000% ~92.000%), the average atmospheric pressure was 877.623 × 100 Pa (871.100 × 100 Pa ~ 884.300 × 100 Pa), the annual highest temperature is 26.798°C (8.700°C~34.300°C), and the annual lowest temperature is 6.833°C (-5.700°C~19.900°C) Table 2.

The results are found in Table 3, where the VIF values of average temperature (°C) and annual lowest temperature (°C) are greater than 10, indicating that there is a multicollinearity between meteorological factors. To overcome the problem of collinearity, LASSO regression is used for meteorological variables screening and selection, the optimal subset is selected by the *C*_*p*_ (Mallow's *C*_*p*_) statistic of the model, five meteorological factors (Table 3) are screened out when *C*_*p*_ = 6.149, then linear regression is performed, and there are four variables that pass the significance test (Table 4): sunshine hours, relative humidity, average atmospheric pressure, and annual highest temperature will have an impact on the number of monthly PTB reports in Guiyang.

### 3.3. ARIMA Model

**Figure 2:**
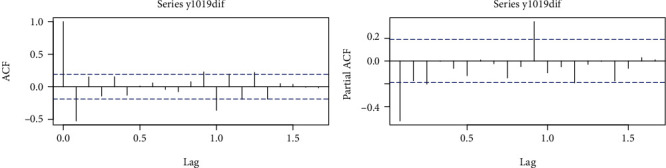
Guiyang PTB monthly registrations after dealing with the 12 step and 1 order difference sequence of ACF and PACF.

We defined the data from 1 January 2010 to 31 December 2019 as the training set to build the prediction model. From Figure 1, we know that the number of monthly PTB reports in Guiyang City has a long-term trend and seasonality; so, the ARIMA (*p*, *d*, *q*) (*P*, *D*, *Q*)_*S*_ model was chosen for fitting. According to the modeling steps, the series was transformed to a smooth series (Dickey − Fuller = −5.855, *p* < 0.01) after a 1st order 12-step difference and conformed to the nonwhite noise property (*p* < 0.01), so that *d* = 1, *D* = 1, and *S* = 12. The parameters *p*, *q*, *P*, and *Q* were estimated by the “auto.arima ()” function was automatically fitted in combination with manual observation of ACF and PACF plots to estimate the parameters *p*, *q*, *P*, and *Q*. The “auto.arima()” function selected ARIMA (1,0,1) (0,1,1)_12_ as the best model, which clearly did not fit the actual situation and required further observation of the ACF and PACF plot (Figure 2), initially identified as *p* = 0 or 1 and *q* = 0 or 1.

**Figure 3:**
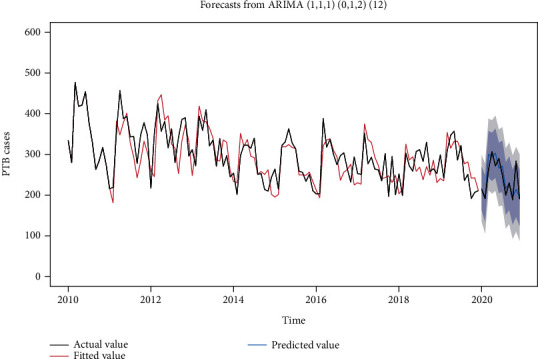
Fitting effect and prediction graph of ARIMA (1, 1, 1) (0, 1, 2)_12_ models.

The identification of *P* and *Q* is more difficult, but in general, it does not exceed order 2; so, we took 0, 1, and 2 from low to high order, respectively, and tried them one by one (Table 5). After considering the fitting effect of each ARIMA model in the training set and the sensitivity of predicting the number of PTB incidence from January 1 to December 31, 2020, we chose the ARIMA (1, 1, 1) (0, 1, 2) _12_ model as the relatively optimal model, which has a white noise series of residuals (*p* = 0.863) and AIC = 1124.440, which is a good fit, see Figure 3.

### 3.4. ARIMAX Model

In order to evaluate the correlation between meteorological factors and the number of PTB registrations in Guiyang with different lags, we developed ARIMA models for sunshine hours, relative humidity, average atmospheric pressure, and annual highest temperature (Table 6) to obtain the residual series of meteorological factors.

**Figure 4:**
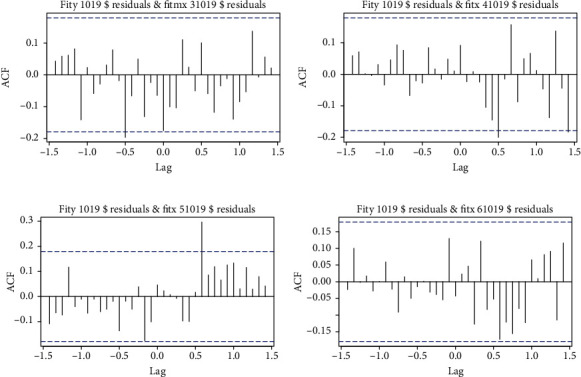
Graph of crosscorrelation functions: (a) sunshine hours, (b) relative humidity, (c) average atmospheric pressure, and (d) annual highest temperature.

**Figure 5:**
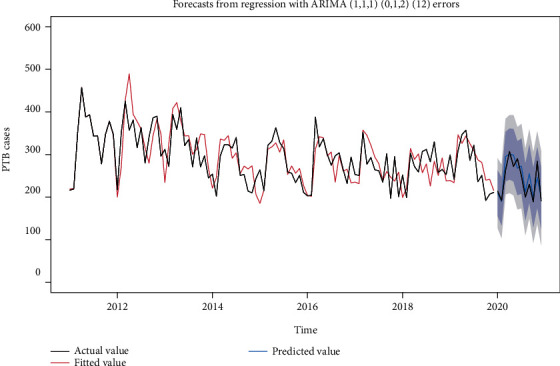
Fitting effect and prediction graph of AAP (7-month lag) + ARIMA (1, 1, 1) (0, 1, 2)_12_ models.

The ARIMA (1, 1, 1) (0, 1, 2)_12_ model for the number of PTB cases in Guiyang City from 1 January 2010 to 1 December 2019 was analyzed using the crosscorrelation function (CCF) with the lag time of the meteorological factor for the same period (Figure 4), and it can be seen from the figure RH (6-month lag) and AAP (7-month lag). Based on this, we combined the RH (6-month lag) and AAP (7-month lag) with the ARIMA (1, 1, 1) (0, 1, 2)_12_ model in turn to construct the ARIMAX model (Table 7). After considering the fitting effect of each ARIMAX model in the training set and the sensitivity of predicting the number of PTB incidences from January 1 to December 31, 2020, we selected the AAP (7-month lag) + ARIMAX (1, 1, 1) (0, 1, 2)_12_ model as the relatively optimal model, which had a white noise series of residuals (*p* = 0.973) and AIC = 998.980, which is a good fit (Figure 5).

### 3.5. Holt-Winters (Additive and Multiplicative) Model

**Figure 6:**
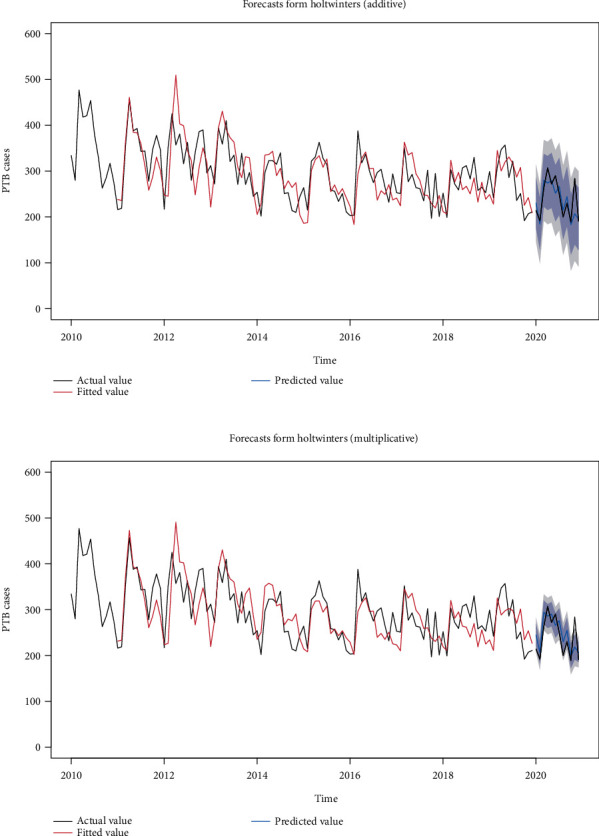
Fitting effect and prediction graph of Holt-Winters (additive and multiplicative) model.

The Holt-Winters (additive) model and Holt-Winters (multiplicative) model were constructed using the Holt-Winters () function in RStudio to automatically fit the data based on the principle of optimality of fit, as the original series has a certain long-term trend and periodicity. The results show that the Holt-Winters (additive) model outperforms the Holt-Winters (multiplicative) model in terms of fitting and prediction sensitivity see Table 8 and Figure 6.

### 3.6. LSTM and Multivariable LSTM Models

**Figure 7:**
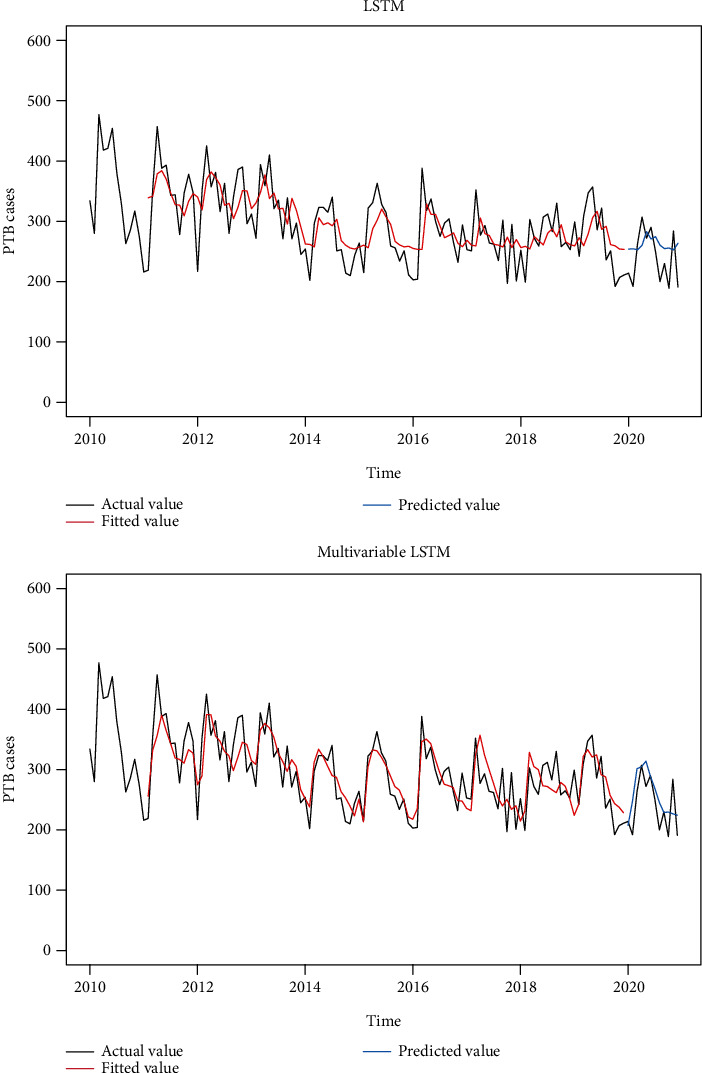
Fitting effect and prediction graph of LSTM and multivariable LSTM+RH (6-month lag) + AAP (7-month lag) models.

Based on the previous analysis, the LSTM model and multivariable LSTM+RH (6-month lag) + AAP (7-month lag) model were constructed according to the model parameters' set in this study. From Figure 7 and Table 9, the multivariable LSTM+RH (6-month lag) + AAP (7-month lag) model outperforms the LSTM in terms of training set fitting and prediction sensitivity.

### 3.7. Comparison of Time Series Model

In this paper, MAPE, RMSE, and MAE were used to assess the fit and predictive sensitivity of the forecasting models (Table 10), and predictive sensitivity (MAPE = 6.901%, RMSE = 20.724, MAE = 17.306) than the ARIMA (1, 1, 1) (0, 1, 12)_12_, Holt-Winters (additive), and multivariable LSTM+RH (lag 6) + APP (lag 7) models performed well. Therefore, the ARIMAX (1, 1, 1) (0, 1, 2)_12_ + AAP (lag 7) model was the best prediction model for predicting the prevalence of TB in Guiyang after the COVID-19.

## 4. Discussion

In recent years, due to the increase in global climate change and extreme weather events, the spread of infectious diseases has been seriously affected, and the existing health problems of human beings have multiplied [33]. In southwest China, the weather usually changes rapidly, with more extreme weather and more susceptibility to meteorological factors. Therefore, it is of great significance to consider the influence of meteorological factors on the occurrence and spread of infectious diseases while constructing mathematical models to predict the epidemic trend of infectious diseases, which will help optimize the prevention strategy of infectious diseases.

In this study, we found that meteorological factors were correlated with the number of PTB reports in Guiyang City from 2010 to 2020 based on monthly PTB reports and meteorological data for the same period, and that there was a lagged effect, with each model significantly improving performance when meteorological factors were included. At the same time, after the decomposition of the multiplicative time series, we found that the number of monthly PTB reports in Guiyang city has the characteristics of seasonality and long-term trend; so. it is a good choice to use the time series analysis technique to construct a prediction model. We chose the classical ARIMA/ARIAMX, Holt-Winters (additive and multiplicative) models and the advanced LSTM/multivariable LSTM models to evaluate the performance of the six models on training set and prediction sensitivity by including or not including meteorological factors. The results of this study showed that the ARIMAX (1, 1, 1) (0, 1, 2)_12_ + AAP (lag 7) model with the inclusion of meteorological factors showed the best performance and was suitable for predicting the epidemic trend of tuberculosis in Guiyang after the occurrence of a COVID-19, and accurately predicting the epidemic trend of tuberculosis could help the local government to formulate appropriate prevention and control measures.

ARIMA and Holt-Winters (additive and multiplicative) models are traditional statistical models for time series prediction, both of which reveal the patterns of historical data over time and are suitable for short-term prediction. They are widely used for predicting the epidemic trends of infectious diseases such as hand, foot, and mouth disease [34], tuberculosis [9], and COVID-19 [35, 36] because of their advantages of easy and fast modeling approach and high prediction accuracy. In this study, the ARIMA model and Holt-Winters (additive and multiplicative) model were generally good predictors and fits for the number of monthly PTB registrations in Guiyang from 2010 to 2020, but the ARIMA model was superior to the Holt-Winters model. The reason for this may be that the ARIMA model constantly adjusts the parameters during the modeling process, taking into account the developmental characteristics of the series, especially for series with complex interactions between long-term trends, periodicity, and stochastic fluctuations. The Holt-Winters model, on the other hand, decomposes the variation pattern of the series by means of exponential smoothing, which is suitable for analyzing data with little variation over time, and its modeling process is simpler than that of the ARIMA model, but the Holt-Winters model wastes serious information on the stochastic fluctuations in the series, which leads to a less than ideal model fitting accuracy.

It is well known that environmental and natural factors largely influence the incidence and transmission of PTB. Using a single time series model may not reflect the changing pattern of PTB incidence; so, it is necessary to develop prediction models containing multiple input variables, while some studies have confirmed that time series prediction models containing influencing factors perform better than single prediction models [37–39]. In a comparison of the ARIMA model with the ARIMAX model, we found that the ARIMAX model with the inclusion of meteorological factors outperformed the single ARIMA model because the ARIMAX model could handle multivariate time series data that included other time series associated with the number of TB reports, so as to improving the predictive accuracy of the model. However, it was also found in our study that the inclusion of more meteorological factors did not imply higher model accuracy, a result similar to that of a time series analysis of PTB conducted in three cities in Jiangsu Province, China [40].

Considering that both the ARIMA and ARIMAX models are linear regression models, we also used a deep learning based LSTM model. The LSTM model captures nonlinear dependencies and performs better on volatile time series with unstable components than the traditional linear ARIMA model and is able to maintain long-term storage of information, thus allowing accurate modelling of data where short-term or long-term dependencies exist [41]. Based on this, one might think that the LSTM model would outperform the ARIMA/ARIMAX and Holt-Winters models in training and prediction, but the univariate LSTM model was found to be the worst performer in our study. The LSTM model is an advanced recurrent neural network, which improves the accuracy of the model prediction results with a larger number of data sets [42]. We then further constructed the multivariable LSTM model, i.e., adding meteorological factors with lagged effects to the LSTM model, and the results obtained showed that the meteorological factors could improve the performance of the LSTM model, but it was still worse than the ARIMA/ARIMAX model and Holt-Winters model. The LSTM model is a complex neural network that requires a large amount of data for training, and too few training samples can lead to overfitting [43]. In this study, we are constructing a time series forecasting model based on monthly data from 2010 to 2019, with a small sample size and strong linear dependence between series; so, the ARIMA/ARIMAX model and Holt-Winters model will perform better when they have a clear trend in the series.

This study compared the predictive performance of ARIMA, ARIMAX, Holt-Winters, LSTM, and multivariable LSTM models with and without meteorological factors using time series analysis techniques, with the aim of finding a suitable model for predicting the trend of tuberculosis epidemic in Guiyang, China. Of course, there are still some shortcomings in this study; firstly, we only considered meteorological factors in our modeling process. In future work, we will incorporate social and economic influences into the prediction model and continuously update the PTB data to obtain more accurate results. Secondly, the monthly TB data we collected did not distinguish between the mobile population and drug-resistant PTB reports, and some of the data were difficult to obtain; so, further research is necessary to predict trends in the mobile population and drug-resistant PTB reports in Guiyang. Finally, we need to further delineate different time scales in future studies to explore the performance of different time series prediction models.

## 5. Conclusions

In this study, we constructed six time series models with and without meteorological factors using monthly PTB reports and meteorological data for the same period from 2010 to 2020 in Guiyang City, China, of which the ARIMAX (1, 1, 1) (0, 1, 2)_12_ + AAP (lag 7) model performed best in the training and validation sets. It shows that the addition of meteorological factors can improve the accuracy of the prediction model, which can be applied to the prediction of the trend of tuberculosis epidemic in Guiyang City, thus helping the local government to formulate effective intervention measures and prevention and control strategies, which is of practical significance and value for the comprehensive prevention and control of TB, and also provide methodological reference for the trend prediction of other infectious diseases.

## Figures and Tables

**Table 1:** The main parameters of the LSTM model and the multivariable LSTM model.

Parameters	LSTM	Multivariable LSTM
Epochs	220	300
Optimization functions	ADAM	ADAM
Learning rate	0.001	0.001
Batch size	30	120

**Table 2:** Monthly meteorological conditions of Guiyang from 2010 to 2020.

Meteorological factors	Mean	Standard deviation	Max.	Min.
Total precipitation (mm)	99.902	92.549	507.100	6.300
Average temperature (°C)	14.752	6.766	24.300	-1.500
Sunshine hours (hour)	86.814	47.013	218.600	2.400
Relative humidity (%)	81.167	5.188	92.000	68.000
Average atmospheric pressure (100 Pa)	877.623	3.764	884.300	871.100
Annual highest temperature (°C)	26.798	5.323	34.300	8.700
Annual lowest temperature (°C)	6.833	7.483	19.900	-5.700

**Table 3:** Results of VIF test and LASSO regression analysis of meteorological factors.

Meteorological factors	VIF value	Regression coefficient
Total precipitation (mm)	2.464	0.000
Average temperature (°C)	40.029	0.000
Sunshine hours (hour)	3.166	-0.748
Relative humidity (%)	2.025	-3.060
Average atmospheric pressure (100 Pa)	3.643	-5.191
Annual highest temperature (°C)	8.044	5.551
Annual lowest temperature (°C)	24.149	-0.889

**Table 4:** Regressor selection results for LASSO.

Parameter	Estimate	Std. error	*t* value	*p* value
Intercept	5492.277	2022.397	2.716	0.008
Sunshine hours (hour)	-0.799	0.173	-4.613	<0.001
Relative humidity (%)	-3.323	1.222	-2.720	0.007
Average atmospheric pressure (100 Pa)	-5.713	2.273	-2.513	0.013
Annual highest temperature (°C)	6.123	1.796	3.409	<0.001
Annual lowest temperature (°C)	-1.352	1.274	-1.062	0.290

**Table 5:** Verification of alternative ARIMA models.

ARIMA models	Training set			Test set			AIC	Box-Ljung test	
	MAPE (%)	RMSE	MAE	MAPE (%)	RMSE	MAE		*X*-squared	*p* value
ARIMA (1, 0, 1) (0, 1, 1)_12_	11.102	39.983	31.659	12.436	32.012	29.046	1131.050	0.301	0.584
ARIMA (0, 1, 1) (0, 1, 0)_12_	12.726	48.733	36.806	13.187	44.787	32.290	1152.190	0.101	0.750
ARIMA (1, 1, 1) (0, 1, 0)_12_	12.712	48.660	36.901	13.665	46.234	33.447	1153.830	0.005	0.942
ARIMA (0, 1, 1) (0, 1, 1)_12_	10.549	39.956	30.731	7.733	26.653	20.055	1122.370	0.105	0.746
ARIMA (1, 1, 0) (0, 1, 1)_12_	11.012	42.498	32.325	7.226	28.546	19.206	1132.510	0.779	0.378
ARIMA (1, 1, 1) (0, 1, 1)_12_	10.462	39.535	30.411	9.936	27.894	24.205	1123.800	0.092	0.762
ARIMA (1, 1, 1) (1, 1, 1)_12_	10.319	38.826	29.973	8.034	26.612	20.406	1124.940	0.048	0.826
ARIMA (0, 1, 1) (0, 1, 2)_12_	10.271	38.819	29.906	7.229	27.913	19.165	1122.680	0.044	0.833
ARIMA (0, 1, 1) (1, 1, 2)_12_	10.307	39.062	30.015	7.179	27.852	19.075	1124.440	0.055	0.814
ARIMA (0, 1, 1) (2, 1, 2)_12_	8.399	32.532	24.309	14.232	41.408	34.847	1115.750	0.001	0.979
ARIMA (1, 1, 1) (0, 1, 2)_12_	10.184	38.375	29.578	7.081	26.771	18.601	1124.440	0.030	0.863
ARIMA (1, 1, 1) (1, 1, 2)_12_	10.277	38.692	29.817	7.302	26.625	19.071	1126.150	0.036	0.849

Note: ARIMA (1, 0, 1) (0, 1, 1)_12_ is the autorecognition model of auto.arima() function.

**Table 6:** ARIMA model for each meteorological factors.

Meteorological factors	ARIMA models	AIC	Box-Ljung test	
			*X*-squared	*p* value
Sunshine hours (SH)	ARIMA (1, 0, 0) (1, 1, 1)_12_	1088.210	0.007	0.932
Relative humidity (RH)	ARIMA (0, 0, 1)	737.420	<0.001	0.998
Average atmospheric pressure (AAP)	ARIMA (0, 0, 0) (0, 1, 1)_12_	364.720	0.379	0.538
Annual highest temperature (AHT)	ARIMA (1, 0, 0) (2, 1, 1)_12_	509.200	0.185	0.667

**Table 7:** Verification of alternative ARIMAX models.

ARIMAX models	Training set			Test set			AIC	Box-Ljung test	
	MAPE (%)	RMSE	MAE	MAPE (%)	RMSE	MAE		*X*-squared	*p* value
RH (lag6) + ARIMAX (1, 1, 1) (0, 1, 2)_12_	10.220	38.076	28.915	10.343	29.498	25.662	995.280	<0.001	0.983
AAP (lag7) + ARIMAX (1, 1, 1) (0, 1, 2)_12_	10.164	37.570	28.511	6.901	20.724	17.306	998.980	0.001	0.973
RH (lag6) + AAP (lag7) + ARIMAX (1, 1, 1) (0, 1, 2)_12_	9.837	36.661	27.819	8.202	24.736	20.429	991.790	0.030	0.862

**Table 8:** Evaluation of fitting and prediction effect of Holt-Winters (additive and multiplicative) models.

Models	Parameter			Training set			Test set		
	*α*	*β*	*γ*	MAPE (%)	RMSE	MAE	MAPE (%)	RMSE	MAE
Holt-Winters (additive)	0.220	0.000	0.480	11.930	42.732	34.438	8.101	28.689	21.003
Holt-Winters (multiplicative)	0.073	0.000	0.396	12.177	43.649	35.323	10.726	29.348	26.041

**Table 9:** The fitting and prediction of LSTM and multivariable LSTM models.

Models	Training set			Test set		
	MAPE (%)	RMSE	MAE	MAPE (%)	RMSE	MAE
LSTM	13.191	44.516	36.250	18.124	44.585	39.176
Multivariable LSTM+RH (lag 6) + AAP (lag 7)	10.105	34.615	28.961	12.758	35.167	28.562

**Table 10:** Evaluation of fitting and prediction effect of time series models.

Models	Training set			Test set		
	MAPE (%)	RMSE	MAE	MAPE (%)	RMSE	MAE
ARIMA (1, 1, 1) (0, 1, 2)_12_	10.184	38.375	29.578	7.081	26.771	18.601
ARIMAX (1, 1, 1) (0, 1, 2)_12_ + AAP (lag 7)	10.164	37.570	28.511	6.901	20.724	17.306
Holt-Winters (additive)	11.930	42.732	34.438	8.101	28.689	21.003
Multivariable LSTM+RH (lag 6) + APP (lag 7)	10.105	34.615	28.961	12.758	35.167	28.562

## Data Availability

The data used and analyzed in this paper are available in the Tuberculosis Management Information System of the China Disease Prevention and Control Information System and the Guiyang Statistical Yearbook.

## Supplementary Materials

Supplementary Materials This supplementary material is the original data for this study and contains monthly data on TB registration reports in Guiyang City from 1 January 2010 to 31 December 2020 and monthly meteorological data for the same period.
