# Supplementary material for: Predicting the Number of Reported Pulmonary Tuberculosis in Guiyang, China, Based on Time Series Analysis Techniques
Source: Comput Math Methods Med. 2022 Oct 30;2022:7828131. doi: 10.1155/2022/7828131 (PMC9637476; doi:10.1155/2022/7828131)
Supplement: Supplementary Materials — This supplementary material is the original data for this study and contains monthly data on TB registration reports in Guiyang City from 1 January 2010 to 31 December 2020 and monthly meteorological data for the same period. [file 7828131.f1.pdf]

time	PTB cases	Total Precipitation (mm)	Average Temperature (°C)	Sunshine Hours (hour)	Relative Humidity (%)
2010/1/1	334	7.1	6.3	63.2	81
2010/2/1	280	6.3	7.7	88	74
2010/3/1	477	23.3	11.5	109	71
2010/4/1	418	57.2	11.3	64.4	80
2010/5/1	421	95	18.3	61	83
2010/6/1	454	189.9	19.4	20.7	87
2010/7/1	380	140.3	23.5	107.3	82
2010/8/1	330	144.1	23.1	185.7	77
2010/9/1	263	156.7	20.1	93.1	85
2010/10/1	285	104.2	14.1	80	85
2010/11/1	317	38.8	11	58.8	82
2010/12/1	275	47.1	6.7	80.3	78
2011/1/1	216	24.7	-1.5	8.3	82
2011/2/1	219	6.5	7.7	59.4	78
2011/3/1	354	22.8	6.8	38.5	79
2011/4/1	457	30.8	14.5	49.2	80
2011/5/1	388	58.9	19	145.2	70
2011/6/1	393	183.5	21.3	56.8	83
2011/7/1	343	31.3	23.3	101.8	74
2011/8/1	344	57.7	23.7	181.2	69
2011/9/1	278	56	20.2	75.1	74
2011/10/1	347	169.7	15.1	38.4	83
2011/11/1	378	71.1	14.3	40.2	81
2011/12/1	347	22.2	3.6	9.9	82
2012/1/1	217	19.6	0.7	4.1	92
2012/2/1	352	24.2	2.5	2.4	91
2012/3/1	425	41	9.6	55.9	82
2012/4/1	357	25.8	17.1	83.8	68
2012/5/1	381	251.1	19.2	34.7	82
2012/6/1	316	211.9	19.8	30.5	88
2012/7/1	363	270	22.8	82.3	85
2012/8/1	280	111	23	134.3	79
2012/9/1	341	135.6	18.2	94.3	83
2012/10/1	386	82.6	15.6	63.6	88
2012/11/1	390	35.6	10.2	44.2	90
2012/12/1	296	18	5.1	51.5	87
2013/1/1	312	11.4	4.2	39.9	83
2013/2/1	272	17.3	8.7	66.1	86
2013/3/1	394	37.9	14.3	116.2	73
2013/4/1	359	43.6	14.8	85.7	81
2013/5/1	410	224.2	18.9	117.5	80
2013/6/1	321	191.7	22.3	171.4	78
2013/7/1	335	264	24	160.5	74
2013/8/1	271	123.7	23.1	148.5	77
2013/9/1	339	81.6	19.5	117.8	79
2013/10/1	271	53.2	14.9	105.1	77
2013/11/1	297	45.9	11.7	51.5	81
2013/12/1	245	31.4	4.9	50.5	76
2014/1/1	254	16.8	6.2	92.1	74
2014/2/1	202	37.7	4.3	32.3	86

2014/3/1	297	89	10.3	54.9	86
2014/4/1	323	46.2	16.4	96.4	83
2014/5/1	323	224.3	18	46.9	83
2014/6/1	315	303.4	21	17.4	88
2014/7/1	340	419	23	121.4	84
2014/8/1	251	167.2	22.7	138.9	79
2014/9/1	253	101.3	21.4	142.8	81
2014/10/1	214	88.3	17.3	132.7	81
2014/11/1	210	55.7	10.6	22.5	89
2014/12/1	244	13	5.3	57.7	77
2015/1/1	264	33.7	6.1	21.7	84
2015/2/1	215	10.9	8.2	63.7	83
2015/3/1	322	31.6	11.3	74.5	86
2015/4/1	331	81.4	16.5	137.7	72
2015/5/1	363	290.3	19.6	100.5	85
2015/6/1	328	353.9	22	59.7	89
2015/7/1	314	116.6	21.9	92.5	81
2015/8/1	259	192	21.5	107.8	85
2015/9/1	256	67.6	19.7	43	89
2015/10/1	234	167.3	16.6	126.4	82
2015/11/1	251	31.7	13.1	73	85
2015/12/1	211	53.9	6	41.8	84
2016/1/1	203	32.1	4.1	21.6	89
2016/2/1	204	10.8	6.3	92	70
2016/3/1	388	90	11.1	76.4	80
2016/4/1	318	163.7	16.5	77.9	82
2016/5/1	337	164.9	19	113.9	80
2016/6/1	300	115.6	22.4	110	80
2016/7/1	275	73	24.3	190.9	77
2016/8/1	297	170.3	23.1	140.2	81
2016/9/1	304	27.1	20.5	102.8	74
2016/10/1	265	108.1	17	93.8	82
2016/11/1	232	59.7	11.2	73.6	84
2016/12/1	294	30.5	8	67.1	80
2017/1/1	253	22	6.6	18.4	87
2017/2/1	251	24.8	7.5	52.3	79
2017/3/1	352	50.6	9.2	33.3	87
2017/4/1	277	74.6	16.5	106.3	75
2017/5/1	293	107.3	18.8	147.2	73
2017/6/1	264	507.1	20.1	65.1	88
2017/7/1	262	123.2	23.5	177.2	78
2017/8/1	235	114.5	23.6	148.4	80
2017/9/1	302	70.5	21.7	93.7	80
2017/10/1	197	41.5	16.4	90.3	82
2017/11/1	295	17.6	11.4	90.4	78
2017/12/1	201	12.2	6.5	78.1	74
2018/1/1	252	57.2	4	15.9	87
2018/2/1	199	15.2	6.3	52.2	74
2018/3/1	303	111.4	13.8	106	75
2018/4/1	272	77	16.7	117.2	74
2018/5/1	259	251.3	20	109.4	80

2018/6/1	307	234.4	21.1	81.7	82
2018/7/1	312	98.4	24.3	169.3	76
2018/8/1	283	145.5	23.2	176.6	78
2018/9/1	330	147.4	20	98.8	82
2018/10/1	258	39.6	13.7	27.3	84
2018/11/1	265	56.5	10.3	83.8	83
2018/12/1	253	19.3	4.6	29.0	86
2019/1/1	299	56.8	4.4	8.8	91
2019/2/1	242	30.0	5.0	35.3	90
2019/3/1	309	45.8	11.2	86.4	79
2019/4/1	347	99.7	17.3	135.2	79
2019/5/1	357	254.0	17.7	73.8	84
2019/6/1	286	249.0	22.0	84.8	83
2019/7/1	322	206.8	22.7	118.6	84
2019/8/1	236	33.7	24.3	218.6	73
2019/9/1	251	130.1	20.7	176.2	74
2019/10/1	192	117.2	16.3	102.4	84
2019/11/1	207	20.3	10.9	64.7	84
2019/12/1	211	10.4	7.2	112.3	90
2020/1/1	214	69.2	6.2	62.9	88
2020/2/1	192	39.5	8.9	63.3	84
2020/3/1	263	22.7	12.4	106.1	80
2020/4/1	307	77.7	13.4	111.2	78
2020/5/1	272	197.8	20.9	178.1	75
2020/6/1	290	213.2	22.4	109.3	83
2020/7/1	250	290.7	23.7	146.4	81
2020/8/1	200	48.0	23.8	190.2	75
2020/9/1	230	288.4	18.8	53.5	90
2020/10/1	189	96.1	14.2	102.5	84
2020/11/1	284	17.8	11.1	133.7	78
2020/12/1	191	16.9	3.3	30.9	86

Average Atmospheric Pressure (100 Pa)	Annual Highest Temperature (℃)	Annual Lowest Temperature (℃)
881.1	21.2	-1.8
876.5	29.2	-2.2
878.2	29.5	-1.6
877.7	28.9	2.5
873.6	29.8	11.3
873.8	29.5	11.8
873.1	31.4	18.4
875.7	31.7	12.7
877.2	32.5	10.9
881.5	23.8	3.2
882.5	21.2	4.2
879.5	17.7	-4.1
882.7	8.7	-5.4
877	26.5	-3.2
881.2	23.5	0.5
877.5	31.6	3
874.8	33.1	10.5
871.5	30.5	13.7
872	32.4	17.4
874.1	33	15.5
876.8	31.6	9.9
881.3	27.8	6.9
880.7	23.5	4.4
884.3	14.4	-1.4
880.7	11.7	-4.1
878.1	13.9	-3
877.6	27.4	0.4
874.7	31.8	6.5
874.2	29.5	12.1
871.1	30.5	13.7
871.1	30.9	18.6
873.7	31.9	14.5
878.8	29.4	10
880.5	26	8.1
879	22.7	3.4
880.4	23.1	-3.7
881.1	20.1	-5.7
878.6	25.3	-1.2
877.5	27.3	0.9
875.7	30.3	5.2
873.9	30.8	10
872	31.9	13.2
871.7	32.9	19.8
873.3	33.2	17.8
877.9	29.2	9.2
882.3	27.4	7.2
882.3	23.6	3.9
882.8	17.5	-3.2
881.7	20.7	-2.5
877.9	22.2	-5.5

878.4	25.7	2.1
876.4	29.7	8.2
874.6	29.1	10
872.1	29.2	16.5
873.2	32.1	18
874.5	31.9	15.9
876.5	31.2	12.8
881.1	25.1	11.2
881.1	21.5	4.4
884.3	16.7	-1.1
881.9	18.4	-3.5
879.2	23.2	-2.8
878.6	30.6	0.2
877	31	5
873.7	30.2	12
872	30.2	16.6
873.1	32.4	14.2
875	30.2	14.8
877.9	28.3	14
881.6	26	7.8
880.5	25.7	3.9
883.3	19.7	-0.1
881.2	17.3	-4.8
883.4	24	-2.7
878.7	24.8	0.1
874.6	30.3	8.3
874.6	29.6	10.9
873.3	31.2	16.2
872.7	32.9	18.6
873.4	33.7	15.6
877.6	28.7	14.9
879.4	29.6	6.6
881.6	23.9	0.1
882.6	19.8	1.3
881.3	19.9	0.8
881.2	23	-0.4
878.3	24.4	2.6
876.2	30.2	6.4
876.7	29.3	11.1
872.9	28.9	15.8
873.8	32.7	17
873.4	31.9	18.3
876.9	30.6	16.2
881.5	28.9	6.5
882.1	23.8	2.7
884.1	16.7	-0.6
879.4	17.6	-4.5
880.3	22.5	-3
877.6	26.3	3.7
877	29	5.9
874.8	30.2	13.2

872.5	29.3	14.2
871.4	32.9	19.9
872.5	32.1	18.1
877.8	31.1	13.1
882.4	21.1	7.9
881.6	21.9	3.6
882.5	22.3	-4.7
882.5	18.4	-4.3
878.6	24.9	-1.3
877.7	28.1	3.1
875.4	30.9	7.4
875.1	30.9	9.7
871.8	30.0	14.5
871.9	30.6	17.1
873.5	33.8	16.3
879.2	30.8	12.6
881.3	28.1	6.7
882.1	22.4	1.2
882.9	21.8	-0.4
880.0	19.5	-1.1
881.5	22.6	-0.9
877.4	28.3	3.1
880.0	27.4	4.2
874.0	34.3	14.4
871.5	29.2	13.8
871.7	30.7	18.6
873.0	31.8	18.1
876.8	32.0	12.7
881.8	25.8	6.4
883.0	25.0	2.2
883.3	15.3	-3.8
